# Integrating metabolomics and histopathology: A method for metabolite recovery from fixed tissue specimens

**DOI:** 10.1063/4.0001205

**Published:** 2026-05-01

**Authors:** Ivan Vučković, Ryan Meloche, Prasanna K. Mishra, Song Zhang, Petras Dzeja, Maria V. Irazabal, Slobodan Macura

**Affiliations:** 1 Metabolomics Core, Mayo Clinic, Rochester, Minnesota 55905, USA; 2 Division of Cardiovascular Diseases, Mayo Clinic, Rochester, Minnesota 55905, USA; 3 Division of Nephrology and Hypertension, Mayo Clinic, Rochester, Minnesota 55905, USA; 4 Department of Biochemistry and Molecular Biology, Mayo Clinic, Rochester, Minnesota 55905, USA

## Abstract

Combined histopathological and metabolomic analysis of the same tissue specimen can reduce biological variability compared to independent analyses of similar samples. However, conventional workflows are incompatible: tissue fixation leads to metabolite loss through dilution and chemical reactions with fixative, while metabolomic extraction typically destroys the specimen. We propose a method that reconciles these conflicting requirements by enabling metabolite recovery from fixative while preserving tissue for histopathology. In this approach, tissue is initially fixed in a small volume of fixative; an aliquot of the fixative is then collected for nuclear magnetic resonance-based metabolomic analysis before additional fixative is added for standard processing. As a proof-of-concept, mouse kidneys from the same subject were analyzed using two protocols: standard perchloric acid extraction (ensuring complete metabolite recovery but destroying tissue) and metabolite extraction from fixative. Several metabolites were fully recoverable from the fixative, others partially, while some were lost due to chemical reactions. Despite some limitations, this strategy may be useful for analyzing precious specimens, such as human biopsies, that must remain intact for further studies. Moreover, using the same specimen for dual analyses reduces the number of animals required and enhances statistical robustness.

NOMENCLATURE

1D: One-dimensional
ADP: Adenosine diphosphate
AMP: Adenosine monophosphate
ATP: Adenosine triphosphate
CrP: Creatine phosphate
FA: Formaldehyde
FF: Formalin fixed
HR: High resolution
MAS: Magic angle sample spinning
MRI: Magnetic resonance imaging
MS: Mass spectrometry
NAD: Nicotinamide adenine dinucleotide
NBF: Neutral buffered formalin
NMR: Nuclear magnetic resonance
PCA: Perchloric acid
PFA: Paraformaldehyde
PHOS: *O*-Phosphoethanolamine
PME: Phosphomonoesters
TMAO: Trimethylamine N-oxide
UDP-GlcNAc: Uridine diphosphate *N*-acetylglucosamine

## I. INTRODUCTION

NMR (nuclear magnetic resonance) is an excellent modality for studying living systems due to its noninvasive nature; however, *ex vivo* experiments often provide more detailed biochemical information. For instance, while *in vivo* tissue spectroscopy typically identifies about twenty metabolites, *ex vivo* tissue extracts can yield identification of hundreds and quantification of dozens. Designing specific studies therefore requires compromises: *in vivo* experiments are truly nondestructive but offer limited information, whereas destructive *ex vivo* experiments provide far richer biochemical detail.

*Ex vivo* imaging and spectroscopy are most often mutually exclusive. Preparation for one type of analysis typically renders the specimen unsuitable for the other. Tissue processed for *ex vivo* imaging loses metabolites during sample preparation, while metabolomic analysis physically destroys the tissue, precluding subsequent imaging.

In this work, we aim to reconcile these otherwise incompatible approaches by enabling metabolomic analysis while preserving tissue integrity for downstream structural characterization, including histopathology. As a proof-of-concept, paired kidneys from the same wild-type mouse were used to compare two workflows: conventional perchloric acid extraction from frozen tissue, which provides near-complete recovery of water-soluble metabolites but destroys the specimen, and metabolite recovery from fixative during tissue fixation, which preserves the tissue for further processing. By using paired organs from the same animal, biological variability is minimized, allowing a direct assessment of metabolite recovery while maintaining compatibility with standard histopathological workflows. This approach reduces the number of animals required and facilitates more robust correlations between metabolomic data and tissue structure.

## II. SPECIMEN HANDLING IN NMR METABOLOMICS AND STRUCTURAL ANALYSIS

Tissue NMR metabolomics and NMR microscopy have largely evolved as independent techniques, primarily due to technical differences. Metabolomics requires high-resolution NMR spectrometers equipped with advanced shimming and specialized probes, whereas NMR microscopy relies on instruments configured for imaging—typically lacking high-resolution shims but incorporating stronger gradient amplifiers and more sophisticated gradient controllers. These distinct hardware requirements mean that spectrometers and scanners are rarely housed in the same laboratory or even within the same department. As a result, sample preparation and handling protocols for the two NMR modalities are generally incompatible and mutually exclusive.

In the absence of superfusion or perfusion, excised tissue rapidly becomes oxygen-limited, leading to increased reliance on anaerobic metabolic pathways and consequent changes in metabolite concentrations relative to *in vivo* levels. This effect is particularly pronounced for phosphorylated metabolites, which degrade *ex vivo* within minutes.^1,2^ To obtain a high-resolution snapshot of tissue metabolite levels at a specific time point, all chemical reactions must be halted immediately. Rapid freezing^3^ of the tissue sample upon collection effectively stops enzymatic and chemical reactions.^4^ The frozen tissue is then homogenized^5,6^ to maximize extraction yield.^7^

The choice of extraction method depends on the tissue type, the class of molecules of interest (e.g., water-soluble compounds, lipids, polysaccharides), the analytical technique to be used, and the experimenter's experience and preference.^1,2,4,5,8–10^ When the goal is to determine water-soluble metabolites, perchloric acid (PCA) extraction is considered the standard method.^11^

PCA extraction rapidly terminates metabolic reactions and separates low-molecular-weight water-soluble metabolites from proteins and other macromolecules. Typically, tissue metabolic reactions are first halted by freeze-clamping and then fully terminated by enzyme denaturation in PCA extraction, preserving metabolite levels at the moment of rapid freezing. PCA is subsequently neutralized with KHCO_3_, producing insoluble KClO_4_, which is removed by centrifugation.

Polar organic solvents such as methanol, ethanol, acetonitrile, and acetone are widely used—often mixed with water—to extract hydrophilic metabolites, while chloroform is employed for hydrophobic compounds. Compared to PCA extraction, polar organic solvents generally yield higher amounts of small metabolites, though with greater variability.^8^ Moreover, methods using methanol/chloroform/water mixtures are more time-consuming, requiring careful phase separation and additional steps for solvent removal. Combined extraction of polar and lipophilic metabolites can be achieved using biphasic mixtures such as methanol/chloroform/water (4:2.85:4, v/v/v).^11^

While the methods described above were specifically developed for *ex vivo* NMR analysis, tissue fixation is generally performed for purposes unrelated to NMR. The primary goal of fixation is to prevent autolysis and tissue degradation, enabling anatomical and microscopic examination after sectioning. Fixatives are broadly categorized into two types: denaturing (alcohol-based) and cross-linking (formalin-based).^12^ The choice of fixative depends on the objectives of subsequent studies—whether preservation of cellular architecture^13^ or maintenance of gene expression at the cellular level^14^ is more critical. These diverse requirements have led to the development of hundreds of fixative formulations optimized for specific traits.^15^ From an NMR perspective, the presence of fixatives complicates analysis; however, given that NMR often plays a supportive role within a broader investigative framework, even partial metabolomic information can be highly valuable. Most commonly used fixatives are formaldehyde-based,^13^ which will be discussed in detail in Sec. III. Interestingly, the alcohol-based fixative methacarn^14^ is chemically similar to some solvents employed in NMR tissue extraction protocols.^4^

## III. EFFECTS OF TISSUE FIXATION ON METABOLITE PRESERVATION

### A. Chemical basis of formalin fixation

The concept of analyzing metabolites in fixed tissue is relatively new in the NMR field, although it has been explored using other techniques. Numerous methods, primarily based on mass spectrometry (MS), have been developed to recover molecular information from pathology slides where tissue is fixed and paraffin-embedded.^16–19^

In contrast, conventional sample preparation for NMR metabolomic profiling renders tissue unsuitable for subsequent imaging—either because it is pulverized, mechanically damaged during handling, or chemically degraded if left unfixed for extended periods. When the specimen is unique or heterogeneous, obtaining both a metabolite profile and morphological information from the same region of the same specimen is highly advantageous.

Conversely, tissue prepared for imaging is typically exposed to large volumes of fixative, often with multiple specimens immersed in a single bath. This process dilutes and mixes metabolites from all samples. Fixation is essential for long-term preservation of tissue structure and texture, primarily for microscopic examination. The most commonly used fixative is formalin,^13^ which is also compatible with NMR imaging. We have previously described in detail the influence of formalin composition and preparation on the NMR properties of fixed tissue.^20^ Here, we briefly examine the relationship between formalin fixation and metabolite detection.

Formalin is a 37% w/v solution of formaldehyde in water, and the typical fixative used is neutral buffered 10% formalin (NBF). It is important to note that the terms “formalin” and “formaldehyde” are often used interchangeably and that 10% formalin corresponds to approximately 4% formaldehyde. Although formaldehyde is a simple molecule, when dissolved in water, it undergoes a series of reactions, forming a mixture of hydrated formaldehyde (methanediol, also known as methylene glycol or methylene hydrate), glycol polymers (polymethylene glycols or paraformaldehyde, PFA), and disproportionation products such as formic acid and methanol.^21^

Hydration of formaldehyde

$${\text{CH}}_{2}O+H_{2}O\;⇄\text{ HO}-{\text{CH}}_{2}-\text{OH}$$

Polymerization of methylene glycol

$$\text{HO}-{\text{CH}}_{2}-\text{OH}+n\text{ HO}-{\text{CH}}_{2}-\text{OH }⇄\text{ HO}-{\left({\text{CH}}_{2}-O\right)}_{n}-{\text{CH}}_{2}-\text{OH}+n\;H_{2}O$$

Disproportionation (Cannizzaro reaction)

$$2\text{ HO}-{\text{CH}}_{2}-\text{OH}+H_{2}O\;\to \text{ HCOOH}+CH_{3}OH$$

Methylation of methylene glycol

$$\text{HO}-{\text{CH}}_{2}-\text{OH}+CH_{3}OH\;⇄\text{ HO}-{\text{CH}}_{2}-\text{OC}H_{3}+H_{2}O$$

**FIG. 1.**
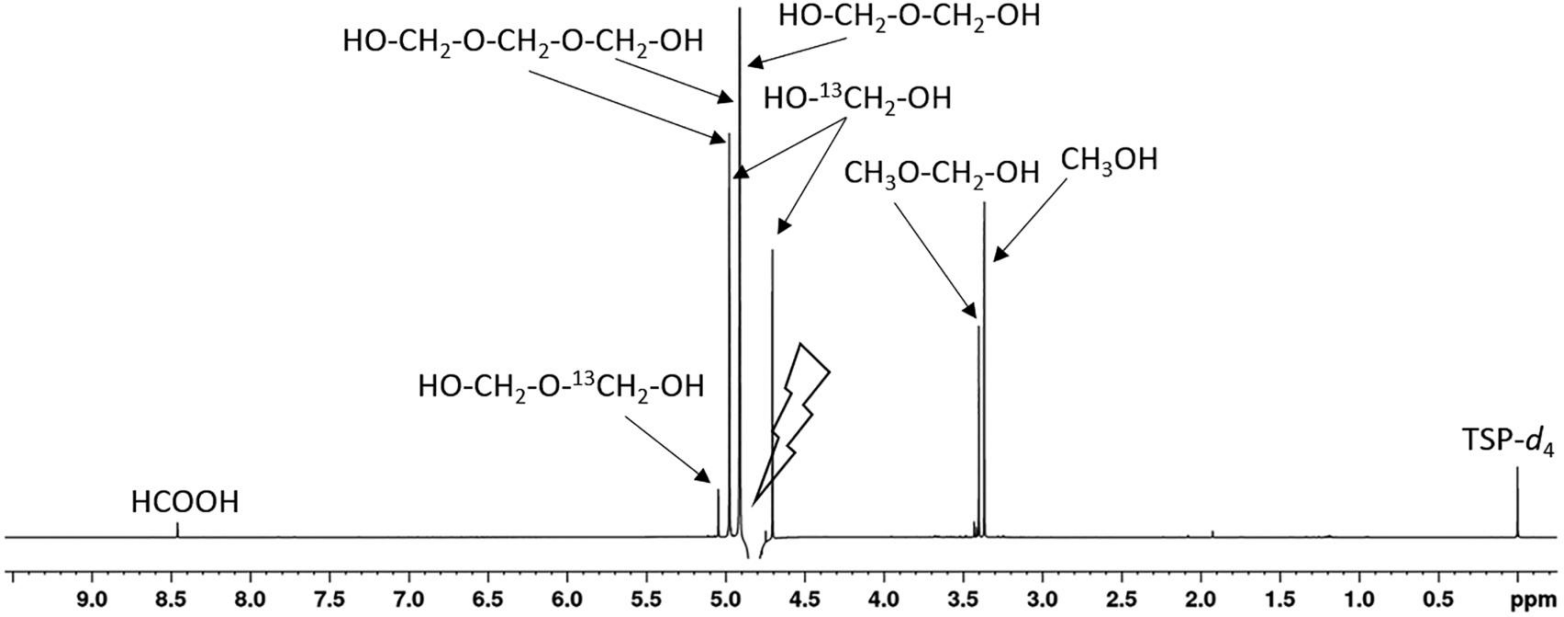
^1^H NMR spectrum of formalin fixative (4% PFA) recorded at 600 MHz in phosphate buffer/D_2_O. Major resonances arising from methylene glycol species, methanol, and formate are indicated. The spectrum was acquired using a 1D NOESY sequence with presaturation, resulting in strong suppression of water and methylene glycol monomer signals.

These reactions are largely reversible, with their rates and equilibrium constants dependent on local conditions such as pH, temperature, concentration, and the presence of salts. Figure 1 shows the ^1^H NMR spectrum of the formalin fixative. Virtually all resonances were identified by comparison with literature data.^22^ The signal of the most abundant component—the methylene glycol monomer—appears near the water resonance and is significantly attenuated during water suppression. The remaining prominent signals correspond to methylene glycol dimer and trimer, ^13^C satellites of methylene glycol, and methoxy groups of methanol and methoxymethanol. Additional signals corresponding to formate and ^13^C satellites of the methylene glycol dimer are also observed.

The primary mechanism of tissue fixation by formalin involves the cross-linking of protein amino (–NH) groups through the formation of methylene bridges:^20^

$$\begin{array}{c} {\text{protein}}_{1}-\text{NH}+{\text{CH}}_{2}O\;\to \;{\text{protein}}_{1}-N-{\text{CH}}_{2}-\text{OH}\\ {\text{protein}}_{1}-N-{\text{CH}}_{2}-\text{OH}+\text{HN}-{\text{protein}}_{2}\;\to \;{\text{protein}}_{1}-N-{\text{CH}}_{2}-N-{\text{protein}}_{2}+H_{2}O \end{array}$$

The reactive –NH group may originate from an exposed protein backbone amide or from a side-chain amino group. Similar cross-linking reactions can also occur with metabolites containing amino functionalities

$$\begin{array}{c} \text{metabolite}-\text{NH}+CH_{2}O\;\to \text{ metabolite}-N-{\text{CH}}_{2}-\text{OH}\\ {\text{metabolite}}_{1}-N-{\text{CH}}_{2}-\text{OH}+\text{HN}-{\text{metabolite}}_{2}\;\to \;{\text{metabolite}}_{1}-N-{\text{CH}}_{2}-N-{\text{metabolite}}_{2}+H_{2}O\;\\ \text{protein}-N-{\text{CH}}_{2}-\text{OH}+\text{HN}-\text{metabolite }\to \text{ protein}-N-{\text{CH}}_{2}-N-\text{metabolite}+H_{2}O \end{array}$$

Because all formaldehyde species exist in dynamic equilibrium, depletion of monomers in solution drives depolymerization of polymers. Consequently, cross-linking ultimately occurs; however, the overall fixation rate depends on the relative concentrations of these migrating species, which differ in both depolymerization kinetics and diffusion rates (with polymers diffusing more slowly than monomers).

### B. Diffusion, dilution, and fixation kinetics

It has been shown that fixing tissue at a fixative-to-tissue volume ratio of 2:1 for 48 h at 20–22 °C is sufficient to ensure proper fixation.^23^ During this process, metabolites present in the tissue gradually leak out, likely reaching a uniform concentration within the fixation vessel. Consequently, the supernatant available for subsequent analysis could contain approximately two-thirds of the original metabolite mass, albeit in a threefold dilution. In modern high-resolution NMR spectrometers, this level of dilution is a minor concern. More critical are the signals originating from the fixative itself, which may overlap with and obscure metabolite resonances, and even more importantly, chemical reactions between the fixative and metabolites containing –NH or –OH groups.

**FIG. 2.**
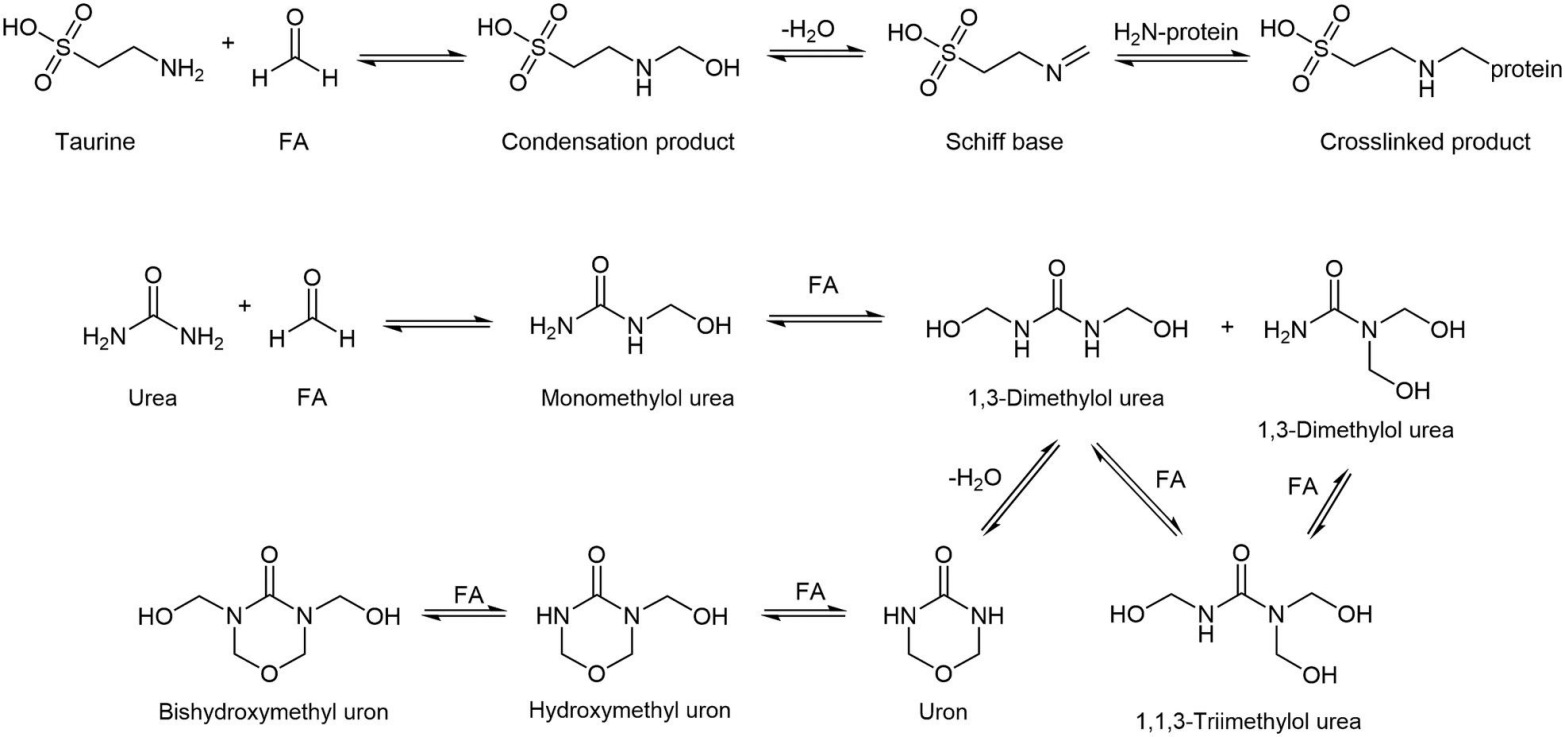
Representative reactions of taurine and urea with formaldehyde species during fixation, including condensation, Schiff base formation, and cross-linking processes.

Such interactions can introduce several changes in the high-resolution spectrum: new signals may appear from fixative components and reaction products, while original metabolite signals may diminish or disappear due to chemical modification. Additionally, unstable metabolites may undergo enzymatic transformations before fixation is complete, and enzymes are fully inactivated. In summary, most metabolites are expected to simply diffuse out of the specimen unchanged, whereas those with reactive –NH or –OH groups may decrease in concentration. For example, taurine and urea^24^ can be partially retained in tissue through cross-linking or chemically modified via reaction with methylene glycol, as illustrated in Fig. 2.

Finally, tissue fixation is a relatively slow process; as the fixative diffuses through the tissue, it becomes locally depleted, resulting in a fixation interval rather than a precise time point. Despite these complications, the simplicity of the extraction procedure and its ability to provide potentially valuable information—otherwise inaccessible—justify further investigation of this approach.

## IV. EXPERIMENTAL

### A. Animals handling and tissue harvesting

Male C57BL/6J mice were obtained from Jackson Laboratories (Bar Harbor, ME) and housed in the Mayo Clinic animal care facility, accredited by the Association for Assessment and Accreditation of Laboratory Animal Care International (AAALAC). All procedures adhered to National Institutes of Health (NIH) guidelines and were approved by the Institutional Animal Care and Use Committee (IACUC) at Mayo Clinic, Rochester. Mice were anesthetized via intraperitoneal injection of ketamine/xylazine. Following laparotomy, the kidneys were exteriorized in the following sequence: the right kidney was harvested after clamping the renal vessels and immediately freeze-clamped in liquid nitrogen using the standard technique,^25^ while the left kidney (∼200 mg) was immersed in 500 *μ*L of formalin fixative (4% paraformaldehyde) and maintained in fixative at room temperature for approximately 1 month prior to analysis.

### B. Tissue fixation and metabolite extraction

The frozen tissue was ground using a liquid nitrogen–cooled tissue pulverizer. Approximately 32 mg of pulverized tissue was homogenized and extracted with 320 *μ*L of ice-cold 0.6 M perchloric acid (PCA). The homogenate was vortexed for 20 s and centrifuged at 10 000 × g for 10 min at 4 °C. The resulting supernatant was neutralized with 100 *μ*L of 2 M KHCO_3_. To 400 *μ*L of the neutralized extract, 100 *μ*L of 0.1 M phosphate buffer and 50 *μ*L of 1 mM TSP-*d*_4_ in D_2_O were added. The mixture was vortexed for 20 s and transferred to a 5-mm NMR tube.

After tissue fixation, 400 *μ*L of the fixative solution was collected. To this, 100 *μ*L of 0.1 M phosphate buffer and 50 *μ*L of 1 mM TSP-*d*_4_ in D_2_O were added. The sample was vortexed for 20 s and transferred to a 5-mm NMR tube.

A formalin fixative reference sample was prepared by mixing 400 *μ*L of 4% paraformaldehyde (PFA), 100 *μ*L of 0.1 M phosphate buffer, and 50 *μ*L of 1 mM TSP-*d*_4_ in D_2_O.

### C. High-resolution NMR spectroscopy

High-resolution ^1^H NMR spectra were acquired on a Bruker 600 MHz spectrometer (Bruker BioSpin, Billerica, MA) equipped with a 5 mm BBI room-temperature probe and an automated refrigerated sample changer (SampleJet). The sample temperature was maintained at 298.2 ± 0.1 K using a variable temperature control unit (BTO 2000).

One-dimensional (1D) ^1^H NMR spectra were recorded with water suppression using the standard NOESY pulse sequence (noesygppr1d; Bruker BioSpin) under the following conditions: 256 scans, 65 536 data points, spectral width of 8403.4 Hz, mixing time of 10 ms, and relaxation delay of 5 s. A line broadening factor of 0.3 Hz (LB = 0.3 Hz) was applied. Spectra were manually phase- and baseline-corrected using TopSpin 3.6 software (Bruker BioSpin).

Processed spectra were analyzed using Chenomx NMR Suite 8.5 (Chenomx Inc., Edmonton, Canada). Compound identification was performed by comparison with the Chenomx 600 MHz Version 11 database and published literature.^26^ Quantification was based on the integral of the internal standard peak (TSP-*d*_4_).

## V. RESULTS

### A. Metabolite spectra

The ^1^H NMR spectrum of the fixative contains only a limited number of resonances, all of which appear as singlets: formate appears at 8.5 ppm, the -CH_2_-O- cluster is located around 4.7 ppm (near the water resonance), and CH_3_-O- signal (methanol) is found at 3.4 ppm. These signals are confined to a narrow spectral region, leaving most of the spectral domain free of fixative-derived contributions.

**FIG. 3.**
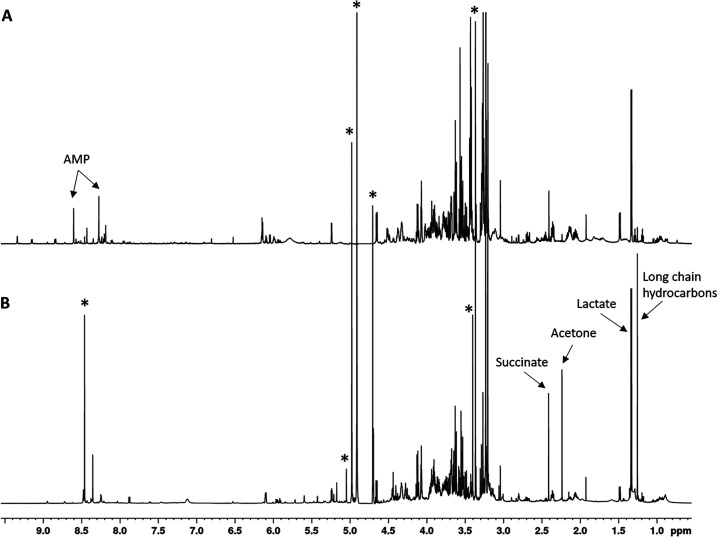
^1^H NMR spectra of frozen tissue extract (a) and fixative supernatant (b). Spectra are shown on the same chemical shift scale for direct comparison. Signals originating from the fixative are marked with asterisks. Selected metabolites are annotated to illustrate differences in composition between the two preparations.

**FIG. 4.**
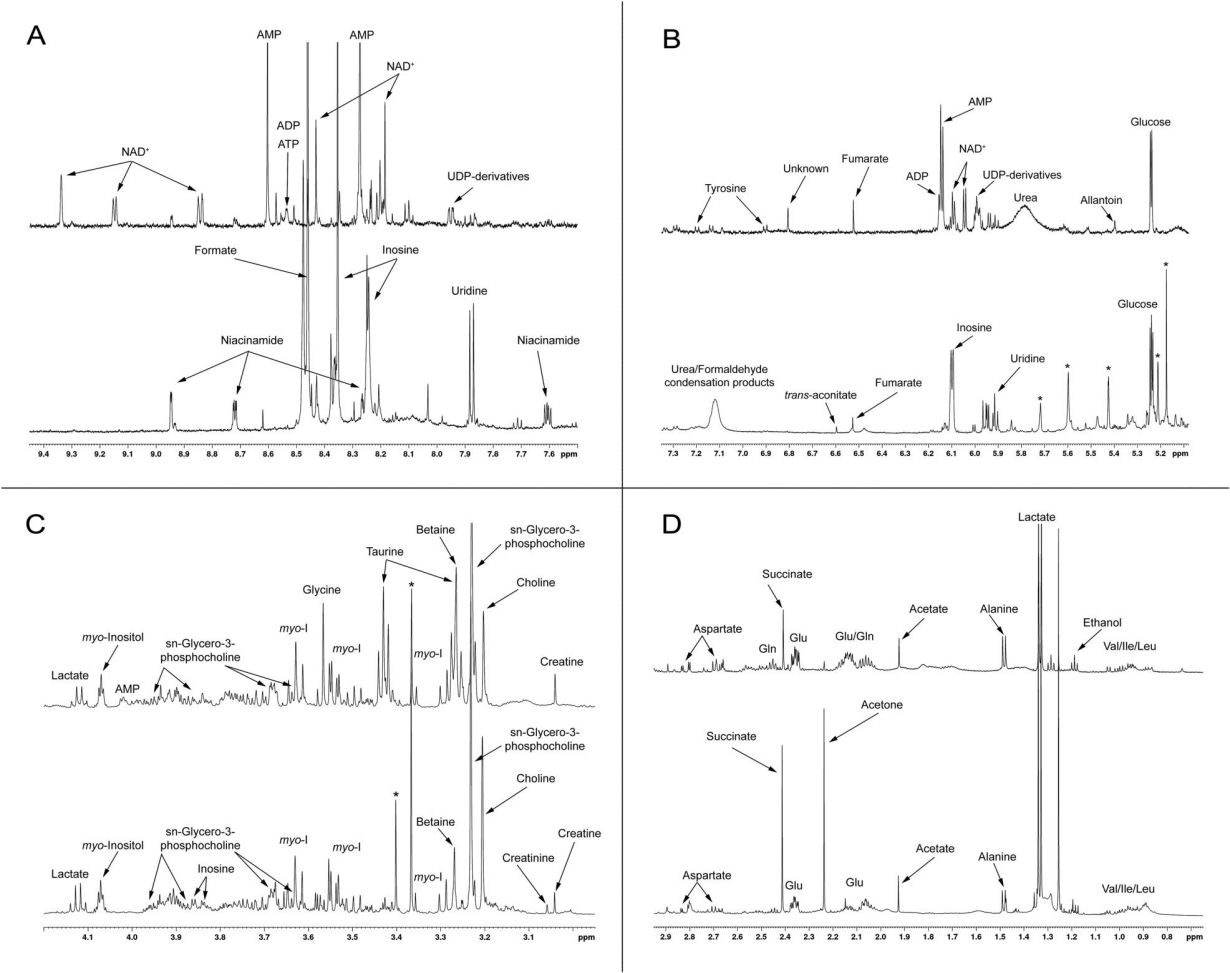
Expanded spectral regions (a)–(d) of ^1^H NMR spectra of frozen tissue extract (top trace) and fixative supernatant (bottom trace). Spectra are scaled according to *myo*-inositol signal intensity. Selected metabolites are annotated to highlight differences in metabolite recovery and fixation-induced transformations between the two preparations.

Figure 3 compares the ^1^H NMR spectra of a frozen tissue PCA extract (panel A) and the corresponding fixative supernatant (panel B). Signals originating from the fixative are marked with asterisks and can be readily identified by comparison with the reference spectrum shown in Fig. 1. While the PCA extract exhibits a rich metabolite profile, the fixative supernatant displays a reduced set of resonances, reflecting differential metabolite recovery during fixation. Expanded views of selected spectral regions are shown in Fig. 4.

**FIG. 5.**
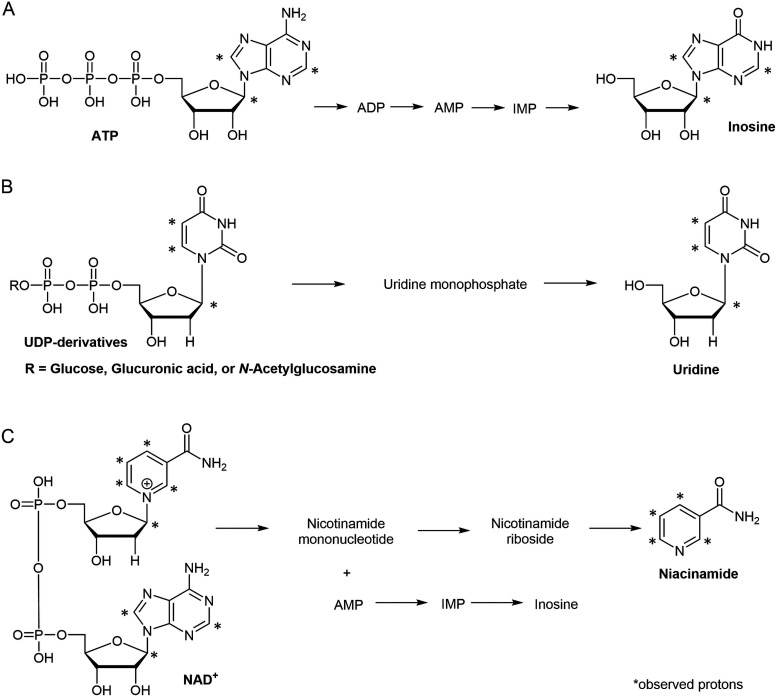
Transformations of selected metabolites during fixation. (a) Degradation of ATP to inosine via intermediate nucleotide species. (b) Conversion of UDP-derivatives to uridine. (c) Degradation of NAD^+^ to niacinamide.

High-energy phosphates (ATP, ADP, and AMP), clearly present in the frozen tissue extract, are absent from the fixative supernatant [Fig. 4(a)]. Instead, inosine is detected, consistent with metabolic changes expected under ischemic conditions.^27^ As summarized in Fig. 5(a), ATP undergoes stepwise degradation to inosine via intermediate nucleotide species. UDP-derivatives are fully converted during fixation to uridine [Fig. 5(b)]. In addition, NAD^+^ is converted to niacinamide through established degradation pathways [Fig. 5(c)].^28^

Several metabolites are absent from the fixative extract, or exhibit markedly reduced recovery, likely due to reactions with formaldehyde, as noted earlier. These include taurine, PHOS, urea, allantoin, and most amino acids. Conversely, a few metabolites are detected in the fixative but not in the PCA extract, such as creatinine (possibly formed by partial conversion of creatine), glycerol, and trans-aconitate.

To compare the extraction efficiency of the two methods, both spectra were normalized to identical myo-inositol signal intensities. myo-Inositol was selected as an internal reference because it is abundant, well-resolved, and chemically stable under fixation conditions, with no evidence of participation in formaldehyde-driven reactions. Based on differences in tissue mass and extraction volumes, an approximate scaling factor of ∼5.25 is expected between the two preparations, assuming complete metabolite recovery and uniform distribution. This value is consistent with the empirically observed scaling required to match myo-inositol signal intensities between spectra (∼5.4), and is further supported by concentration estimates obtained from Chenomx analysis.

Using this normalization framework, the relative intensities of observed metabolites were compared and summarized in Table I. Metabolites are grouped according to their relative abundance in the two extracts based on normalized signal intensities, with additional indication of those detected exclusively in the fixative or in the PCA extract. These categories reflect approximate differences relative to the empirically established reference level, rather than absolute concentration ratios. Importantly, metabolites classified as “high” are not necessarily better recovered, but reflect chemical transformation, degradation, or accumulation of products during fixation.

**TABLE I.** Semi-quantitative comparison of metabolite recovery in fixative supernatant relative to PCA extraction from frozen tissue.

Detected only in fixative		High		Comparable to PCA		Low		Detected only in PCA	
1	Creatinine^a^	4	Acetone	13	Acetate	28	Aspartate^e^	37	ADP^f^
2	Glycerol	5	Choline	14	Alanine	29	Fumarate	38	AMP^f^
3	*trans*-Aconitate	6	Formate^b^	15	Betaine	30	Glutamate^e^	39	ATP^f^
		7	Inosine	16	Carnitine	31	Glycine^e^	40	Allantoin^e^
		8	Lactate	17	Creatine	32	Histidine^e^	41	Glutamine
		9	Niacinamide^c^	18	Glucose	33	Malate	42	Glutathione
		10	Pyruvate	19	Isoleucine	34	Phenylalanine^e^	43	IMP^f^
		11	Succinate	20	Leucine	35	TMAO	44	NAD^+^^g^
		12	Uridine^d^	21	Lysine	36	Tyrosine^e^	45	PHOS^e^
				22	*O*-Acetylcarnitine			46	Taurine^e^
				23	*O*-Phosphocholine			47	UDP-GlcNAc^h^
				24	Valine			48	UDP-glucose^h^
				25	*myo*-Inositol			49	UDP-glucuronate^h^
				26	*scyllo*-Inositol			50	UMP^h^
				27	*sn*-Glycero-3-PC			51	Urea^e^

^a^ From creatine

^b^ From fixative

^c^ Degradation of NAD^+^

^d^ Degradation of UDP

^e^ Reaction with FA

^f^ Degradation to inosine

^g^ Degradation to niacinamide

^h^ Degradation to uridine

## VI. DISCUSSION

In this work, we aimed to expand the repertoire of *ex vivo* NMR methods to include fixed tissue by reconciling two traditionally incompatible specimen preparation protocols: metabolomic spectroscopy and downstream structural analysis. Tissue fixation was originally developed to prevent cellular degradation and preserve samples for long-term storage. In NMR microscopy of tissue (e.g., biopsies, postmortem samples, small animal organs), specimens are typically fixed, and during this process, metabolomic information is largely lost. Conversely, retrieving metabolites requires destructive extraction, rendering the specimen unavailable for imaging.^29^

NMR metabolomics of the fixative in equilibrium with the tissue is inherently imperfect. First, fixation dilutes metabolites by increasing the total volume (tissue plus fixative). Second, fixatives chemically interact with metabolites containing –NH or –NH_2_ groups, either transforming them or rendering them undetectable by cross-linking to tissue proteins. Third, formalin fixative introduces its own spectral signals (Fig. 3), which can overlap with metabolite resonances. In addition, the inherently slow nature of tissue fixation may limit accurate representation of the *in vivo* metabolic state, as post-excision metabolic changes and brief ischemic periods can occur prior to complete fixation. However, several metabolites remain stable following short ischemic intervals, suggesting that meaningful metabolic information can still be preserved under delayed processing conditions.^30^ Despite these limitations, metabolomics of fixed tissue can still provide valuable information, as many metabolites of interest remain detectable under fixation conditions (Table I). Importantly, this approach enables acquisition of both metabolomic and imaging data from the same specimen.

Figures 3 and 4 clearly demonstrate that spectra obtained from the same tissue using two different extraction protocols differ substantially. These differences arise from variations in extraction efficiency, introduction of new chemical species from the fixative, chemical reactions between fixative and certain metabolites, and incomplete quenching of enzymatic activity during fixation. To interpret these differences, it is essential to consider chemical modifications occurring under fixation conditions. First, fixation requires time for the fixative to penetrate tissue and halt enzymatic reactions such as glycolysis. Even after biochemical reactions cease, fixatives do not prevent hydrolysis of phosphorylated metabolites. It is well established that phosphorylated compounds—particularly ATP and creatine phosphate—spontaneously hydrolyze into inorganic phosphate and their respective substrates, a process catalyzed by divalent cations (Ca^2+^, Mg^2+^) naturally present in tissue. Consequently, phosphorylated metabolites are completely absent from the fixative supernatant. Similarly, relatively stable metabolites such as taurine, PHOS, urea, and certain amino acids (Table I) are also absent, likely due to chemical reactions with fixative components. These compounds contain free amino groups that react with methylene glycol and eventually become cross-linked, as described earlier. Nevertheless, as shown in Table I, many metabolites are recovered with comparable efficiency using both extraction methods.

A major limitation of the present study is that it was conducted on a small number of samples from a single animal. Therefore, this work should be viewed primarily as a proof-of-concept demonstrating the feasibility of metabolomics analysis from fixed tissue, rather than as a comprehensive protocol. Nonetheless, given that specimen preparation must always be tailored to the specific tissue type and research question, the analysis presented here provides a valuable foundation for future investigations.

Fixation time represents an important methodological variable. Although the present experiments employed a prolonged fixation period (∼1 month), literature reports indicate that formalin fixation is typically complete within 24–48 h under standard conditions.^23^ This suggests that the conditions used here reflect a post-fixation state rather than ongoing fixation. The use of prolonged fixation also reflects the practical scenario of analyzing archived fixative solutions, potentially extending the applicability of this approach to existing specimen collections. In additional experiments using shorter fixation times (e.g., 72 h), we observed qualitatively similar metabolite recovery and spectral profiles. A systematic evaluation of fixation time effects on metabolite recovery, stability, and quantification remains to be established.

Several practical considerations may improve the consistency and interpretability of results when applying this approach. Formalin remains a pragmatic choice due to its widespread use, compatibility with routine histopathology, and relatively simple and well-characterized spectral background. To limit dilution effects while maintaining effective fixation, the fixative-to-tissue ratio should be kept low and consistent (e.g., 2:1), with fixation times in the range of 48–72 h serving as a practical starting point under controlled conditions. Tissue size and handling should be standardized as much as possible, and specimens should be fixed individually to avoid metabolite exchange between samples. Spectral regions dominated by fixative-derived signals should be excluded or treated with caution, including formate (8.46 ppm), methanol and methoxymethanol (3.36–3.42 ppm), and the broadened water/methylene glycol region (4.6–5.2 ppm). In addition, fixation aliquots should be collected prior to any further processing, and spectra should be acquired and processed using a consistent workflow across samples.

Alternative fixatives may offer advantages for metabolomics applications. For example, methacarn—an older methanol-based fixative that has recently regained attention for preserving gene expression in pathology slides^14^—may provide improved metabolite recovery due to reduced chemical reactivity compared to formalin. However, when combining metabolomic analysis with imaging of the same specimen, the choice of fixative inevitably involves a compromise between structural preservation and metabolite recovery.

Another promising direction is the application of this concept to high-resolution magic angle spinning (HR-MAS) NMR of intact tissue or tissue homogenates. In HR-MAS experiments, sample sizes are small, allowing fixative to diffuse throughout the tissue within minutes, or even faster in homogenized samples. Immediately after harvesting, tissue could be exposed to a limited amount of fixative (not necessarily formalin) to rapidly halt enzymatic activity. This approach would enable experiments to be performed at room temperature while maintaining chemical stability of the specimen during extended NMR measurements, including both 1D and 2D acquisitions under HR-MAS conditions.

## VII. CONCLUSION

To broaden the applicability of *ex vivo* NMR to heterogeneous and valuable specimens that require subsequent imaging (e.g., human biopsies),^29^ we investigated metabolomic profiles obtained from the fixative supernatant. Although this approach is not without limitations, the analysis of the supernatant provides access to metabolite information that would otherwise be lost during routine tissue fixation. This proof-of-concept demonstrates that metabolomics of fixed tissue can complement imaging studies, enabling dual analysis from the same specimen and reducing biological variability in integrated investigations.

## Data Availability

The data that support the findings of this study are available from the corresponding author upon reasonable request.
